# Police Violence in Health Care Settings in US Media Coverage

**DOI:** 10.1001/jamanetworkopen.2023.42998

**Published:** 2023-11-13

**Authors:** Altaf Saadi, Victor E. Ray

**Affiliations:** 1 Department of Neurology, Massachusetts General Hospital, Harvard Medical School, Boston; 2 Department of Sociology and Criminology, University of Iowa, Iowa City

## Abstract

**Importance:**

Hospitals do not collect or share data tracking their policing and security activities despite their reliance on police and security personnel, including armed officers. Thus, little is known about how hospital security is associated with patient and staff safety.

**Objective:**

To examine the harms exerted by health care–affiliated police and security personnel.

**Design, Setting, and Participants:**

For this qualitative study, data were collected using a systematic Media Cloud search for US news media coverage from January 2011 to May 2022. A total of 18 987 articles on policing and hospitals were screened and a content and thematic analysis of articles that met the search criteria was conducted, which involved incidents that revealed harm to patients, patients’ families, and staff. Data were analyzed from October 2022 to April 2023.

**Main Outcomes and Measures:**

Incident year, incident location (hospital name, city, state), survivor and victim characteristics (race and ethnicity, presence of mental illness), and a narrative description of the incident focusing on outcomes of harm exerted by police and security personnel in the health care setting.

**Results:**

A total of 48 unique stories across 25 US states were included. The median (range) year published was 2017 (2009-2022). Harms reported to have been perpetuated by health care–affiliated police and security personnel were identified within 5 domains from 48 unique incidents: (1) patients shot by police or security personnel (17 patients); (2) patients subject to excessive use of force (17 patients); (3) patients arrested (7 patients); (4) patients subject to sexual assault (2 patients); and (5) hospital personnel or those considered collateral damage shot, injured, or arrested (5 individuals). Most survivors and victims were Black, although the race and/or ethnicity of involved individuals was not routinely reported across the news stories. Mental illness was the most documented medical condition among patients injured or killed by health care–affiliated police and security personnel.

**Conclusions and Relevance:**

This qualitative study of US news media found that police and security personnel in hospitals were reported to have perpetuated harm via excessive force, sexual assaults, injuring patients and health care workers, and fatal shootings. Compounded by a lack of transparency and accountability mechanisms, this may represent an underrecognized manifestation of structural racism at the organizational level. Policy suggestions include introducing accountability measures, deescalation techniques, and removing arms from hospital security personnel to reduce harm and fulfill health care’s healing mission.

## Key Points

**Question:**

What are the harms of policing in health care settings?

**Findings:**

In this qualitative study, analysis of 48 unique stories in US news media revealed harms across the following 5 domains: (1) patients shot by police or security personnel; (2) patients subject to excessive use of force; (3) patients arrested; (4) patients subject to sexual assault; and (5) hospital personnel or those considered collateral damage shot, injured, or arrested. Most survivors and victims were Black and had mental illness.

**Meaning:**

These findings suggest that health care organizations’ reliance on police and security personnel can exert harm on patients and staff, with potential for disproportionate outcomes for racially marginalized people and those with mental illness.

## Teaser

This qualitative study evaluates the harms exerted by health care–affiliated police and security personnel as reported in US media coverage.

## Introduction

Police violence is increasingly recognized as an unaddressed public health crisis in the US. A growing literature shows exposure to police violence is associated with adverse health consequences, including medical mistrust and poor mental health beyond co-occurring forms of trauma and violence exposure.^1,2^ Police violence, alongside overpolicing in the lives of persons marginalized by racist and oppressive structures,^3^ is an acute and chronic stressor impacting health and exacerbating health disparities. Yet medicine and public health literature is largely silent about the presence and consequence of police in hospitals, although at least 29 states allow hospitals to form their own police departments, and hospitals that do not employ police officers often employ security personnel.^4^

Health care organizations are not required to report encounters with their police or security staff, so little data are available on law enforcement operations in health care settings. High-profile news stories have often highlighted how police and security personnel employed in health care inflict harms against minoritized individuals, such as the story of a Black student who was shot by hospital police while admitted to the hospital for treatment of bipolar disorder.^5^

Such stories highlight the dangers to patients that police in hospitals pose, especially as police and armed security personnel are becoming increasingly common. In a recent national survey of members of a health care security and safety association spanning 340 US hospitals, 72% of hospitals employed nonsworn security personnel and 21% employed police officers.^6^ Regardless of whether they were designated as police, officers had a variety of tools at their disposal: handcuffs (96%), batons (56%), hand guns (52%), and electrical weapons such as tasers (47%). Eighty-eight percent of hospitals had security personnel who were permitted to handcuff, and about half had security personnel authorized to issue citations or arrest patients, visitors, or nonadmitted patients.^6^ In this way, hospital security forces are purveyors of law enforcement without official state sanction. Compared with previous studies, the study showed a rise in armed security. Yet despite this increase, little is known about how increased security in hospitals, particularly armed security, is associated with patient safety.

We present a content and thematic analysis of US media coverage that outlines the ways in which police or security personnel in hospitals can exert harm to patients and hospital workforce. Because of the lack of data tracking police violence in health care settings, this article relies on media reports and is not an attempt to be systematic. The goal is to highlight potential harms from policing in hospitals in hopes of spurring systematic data collection and potential policy interventions.

## Methods

We conducted a qualitative study with content and thematic analysis of news media coverage in the US from January 2011 to May 2022 to examine the practice of police or security personnel deployment in health care organizations. We analyzed data from October 2022 to April 2023. Per the Common Rule, this analysis did not require approval or the need for informed consent by the Massachusetts General Hospital institutional review board because no individual patient data were used for analysis. The study was performed in accordance with the Standards for Reporting Qualitative Research (SRQR) reporting guideline.

### Data Analysis

We used Media Cloud,^7^ an open-source platform that allows researchers to analyze media coverage of a particular topic over time. Our search terms were “hospital security,” “hospital guard,” or “hospital police” AND “violen* OR aggress* OR force* OR injur* OR assault OR harm*,” and we set a parameter that the search terms be within a distance of 2 to 3 words from each other. We arrived at this parameter through a series of tests for which we set distances of 50, 20, 10, 5, 3, and 2 words between search terms, and then we took 50 articles from each search output to review each article to determine whether it covered deployment of police or security personnel in a US health care setting. We found that the greater distance resulted in too many irrelevant articles, identifying articles that covered police and security personnel outside health care settings.

Two individuals (A.S. and another reviewer) screened 100 articles from the final search term output to determine if an article should be included for analysis. There was 100% agreement, demonstrating strong interrater reliability. Subsequently, articles were screened by the first author (A.S.). Articles were included if they referenced the actions of police or security personnel in US health care settings. This included hospital or municipal police because of prior ethnographic evidence suggesting potential for blurring of their roles and presence of municipal police requiring implicit or explicit approval by the health care organization.^8^ Two individuals (A.S. and another reviewer) independently extracted information from relevant articles, including incident year, incident location (hospital name, city, state), survivor and victim characteristics (race and ethnicity, presence of mental illness), and a narrative description of the incident. There was 100% agreement. Data about race and ethnicity were collected due to existing evidence about racial disparities in policing practices. We used Excel version 16.40 (Microsoft) for data management. The screening and coding were conducted by the first author (A.S.), and a paid, trained research assistant. Three more incidents were identified by manual searches using an online search engine and the same search terms used for the Media Cloud analysis to ensure all relevant media articles were included. One unique incident could have multiple articles written about it; for example, if different articles were written to capture the evolution of a legal case through litigation or settlement processes. One unique incident could have also included multiple survivors or victims, especially if there was both patient and collateral damage harm (see Results). For all incidents, we relied on more than 1 news article to maximize information gathered during data extraction. We then used summative content analysis^9^ and inductive thematic analysis to group incidents into related themes regarding police and security personnel actions in health care settings.^10^ Taking an inductive approach to thematic analysis allowed themes to emerge rather than imposing preconceived ideas.

## Results

A total of 18 987 articles from January 2011 to May 2022 were reviewed, with 48 unique incidents included and analyzed for content and thematic analysis. We found that harms reported to have been perpetuated by health care–affiliated police and security personnel resulted in harms across 5 themes: (1) patients shot by police or security personnel (17 patients); (2) patients subject to excessive use of force (17 patients); (3) patients arrested (7 patients); (4) patients subject to sexual assault (2 patients); and (5) hospital personnel or collateral damage shot, injured, or arrested (5 individuals) (see Table for description of news reports).^11,12,13,14,15,16,17,18,19,20,21,22,23,24,25,26,27,29,30,31,32,33,34,36,37,38,39,40,41,44,45,46,47,49,52^ These themes are not mutually exclusive. For example, in an incident involving a young Black man who was arrested while visiting his newborn in the neonatal unit, his newborn sustained a skull fracture amid the altercation with police officers, with this incident representing harms across 2 themes.^55^

**Table.** Patient and Health Care Personnel Harms From Law Enforcement in Health Care Settings

Incident year	Incident location		Characteristics of survivor(s) and victim(s)				Brief narrative description of event
	Hospital	City, state	Age	Gender	Race and ethnicity	Mental illness	
**Patients shot by police officers**							
2013	Children’s Hospital of Wisconsin	Wauwatosa, Wisconsin	22	Male	Black	NA	Man was shot while fleeing arrest from police in the hospital, where he was visiting his baby in the neonatal unit. In the altercation with police, the baby sustained a skull fracture.^10^
2013	Massachusetts Eye and Ear Infirmary	Boston, Massachusetts	36	Male	White	NA	Incarcerated patient reached for an officer’s gun when his handcuffs were removed, resulting in another officer shooting the man in the chest.^11^
2015	Marymount Hospital	Garfield Heights, Ohio	NA	NA	NA	Y	Altercation between police occurred while patient was undergoing psychiatric evaluation, resulting in patient being tased and shot in the back of the neck by an off-duty officer. Both local police and hospital police were involved.^12^
2015	Harbor-UCLA Medical Center	Torrance, California	26	Male	Hispanic	Y	Incarcerated patient with bipolar disorder reached for an officer’s gun when his handcuffs were briefly taken off, prompting an officer to shoot him.^13^
2015	St Cloud Hospital	St Cloud, Minnesota	50	Male	NA	NA	Patient was shot with a stun gun by hospital security after the patient took a gun from a county deputy and shot the deputy. The patient went into cardiac arrest and died.^14^
2016	Centra’s Lynchburg General Hospital	Lynchburg, Virginia	28	Male	White	Y	Altercation between police occurred while patient with bipolar illness was undergoing psychiatric evaluation and had become upset and anxious after hours of waiting. The patient disarmed an armed security guard of his stun gun and was shot 4 times in the back, leaving the patient paralyzed.^15^
2016	St Joseph Medical Center	Houston, Texas	26	Male	Black	Y	Patient with bipolar disorder admitted to the psychiatry unit was shocked with a taser and shot in the chest by 2 off-duty police officers moonlighting as security guards, who arrived when a nurse summoned security because the patient was refusing to tie his gown.^5^
2017	University Medical Center	Las Vegas, Nevada	NA	Male	White	Y	Patient with mental illness and suicidality pointed a stun gun at a nurse and security guard after taking the stun gun from an unattended bag left by the guard. Patient was subsequently shot by a police officer.^16^
2018	Orlando Regional Medical Center	Orlando, Florida	33	Male	White	Y	Patient with mental illness being evaluated for chest pain and anxiety attacks threatened to “shoot anyone who came near him.” Officers approached him, he appeared to reach for a gun, and 3 officers shot him, although it was later determined patient was unarmed.^17^
2019	Cape Fear Valley Hospital	Fayetteville, North Carolina	NA	Male	Black	Y	Patient under arrest was shot dead after trying to grab the gun of the police officer accompanying him in the hospital.^18^
2020	Dallas VA Medical Center	Dallas, Texas	NA	Male	White	Y	Patient with mental illness including schizophrenia and bipolar disorder was shot and killed by 2 hospital police after he tried to attack them with a knife.^19^
2020	Community Hospital	Munster, Indiana	22	Male	Black	Y	Patient admitted to psychiatric ward attacked a nurse and disarmed an armed security guard, resulting in the patient being shot and killed by another armed security guard.^20^
2020	Harbor-UCLA Medical Center	Torrance, California	38	Male	Hispanic	Y	Patient in mental health crisis shattered a window in his room with a medical device and then attempted to enter a nearby room, resulting in him being shot 7 times and killed by a county deputy.^21^
2021	Mount Carmel St Ann’s Hospital	Westerville, Ohio	27	Male	Black	Y	Patient with mental illness was killed after a struggle with police who discovered he had a gun concealed in his pants.^22^
2022	Duke University Hospital	Durham, North Carolina	38	Male	Black	Y	Patient with substance use disorder attempted to reach for police officer’s gun and was subsequently shot and killed.^23^
2022	Cleveland Clinic Indian River Hospital	Vero Beach, Florida	29	Male	White	Y	Patient in the ED after attempting suicide was shot and killed by 2 officers after running through the hospital with a pair of scissors and raising them in the direction of police.^24^
2022	Baylor Scott and White Medical Center	Irving, Texas	34	Male	Black	NA	Patient in the ED for medical reason was noted by hospital staff to display “unusual behavior” and have a firearm, prompting them to involve a police officer. Both hospital and city police were called, with at least 5 officers arriving on the scene. The patient fired his gun and was then shot and killed by the officers.^25^
**Patients subject to excessive use of force**							
2009	Bridgewater State Hospital	Bridgewater, Massachusetts	NA	Male	White	Y	Patient with paranoid schizophrenia died of a heart attack when strapped to his bed by security guards not trained on appropriate restraining techniques.^26^
2010	Cincinnati’s University Hospital	Cincinnati, Ohio	45	Male	Black	Y	Patient with bipolar disorder hospitalized for a psychotic episode was dragged into a seclusion room by at least 7 hospital officers who tased him multiple times and placed him in restraints. He subsequently experienced a cardiac arrest and died.^27^
2011	Yale-New Haven Hospital	New Haven, Connecticut	53	Male	Black	NA	Patient in ED with his daughter for medical evaluation was preparing to leave when he was approached by officers who pushed him; threw him to the ground, resulting in concussion and rotator cuff injury; and restrained him. His daughter, who attempted to protect him, was also physically restrained by staff.^28^
2014	Jackson Park Hospital and Medical Center	Chicago, Illinois	24	Male	Black	Y	Hospital police were seen on surveillance footage punching and shoving a patient undergoing mental health evaluation, who was restrained and handcuffed.^29^
2015	MedStar Washington Hospital Center	Washington, DC	74	Male	NA	NA	Patient left the hospital without being discharged, was escorted back to the hospital by 2 guards with whom there was an altercation. The man subsequently died of “blunt force injuries.”^30^
2016	VA hospital	El Paso, Texas	70	Male	Hispanic	NA	Patient had an altercation with 2 hospital security guards and was placed in a chokehold and beaten, experiencing shoulder injury, ear and hand pain, and difficulty swallowing following the incident.^31^
2017	Napa State Hospital	Napa, California	64	Male	Hispanic	Y	Patient with bipolar disorder was upset and shouting obscenities. A staff member pushed her personal alarm, prompting 4 officers to arrive, with 1 slamming the patient’s face into a concrete wall resulting in a fractured eye socket, concussion, broken teeth, and facial cuts.^32^
2017	Largo Medical Center Indian Rocks Road Campus	Largo, Florida	27	Male	Black	NA	Patient was noted to be combative with hospital staff, prompting hospital staff to call police. When approximately 7 to 9 police officers arrived on the scene, patient subsequently fought with officers and they tased him multiple times and restrained him. He subsequently went into cardiac arrest and died.^33^
2017	Grant Medical Center	Columbus, Ohio	38	Male	Black	Y	Man visiting a family member that worked at the hospital was hit with a baton, pepper sprayed, and forced to the ground by 2 hospital officers and a security guard. He allegedly swung at security personnel when he was leaving the hospital.^34^
2018	Kansas City VA Medical Center	Kansas City, Kansas	66	Male	NA	NA	Patient was stopped by VA police because he was going the wrong way in the parking lot. In altercation that ensued, the patient was tackled, experienced a traumatic brain injury, and died 2 days later.^35^
2018	Hospital	Mesa, Arizona	23	Male	Hispanic	NA	Patient was transported to a hospital due to injuries he sustained from police violence outside the hospital. In the ED, patient tried to flee his room but was pushed back by officers who threw him onto a wall, gouged him in the eye, and struck his head with a large flashlight.^36^
2018	Advocate Christ Medical Center	Dolton, Ilinois	51	Male	NA	NA	Altercation with hospital security led patient to be shot with a stun gun, restrained facedown with handcuffs by hospital security guards, and injected with sedatives by medical staff. He subsequently died.^37^
2018	Detroit Receiving Hospital	Detroit, Michigan	NA	Male	Black	Y	Patient with mental illness in hospital for a mental health crisis was apprehended by hospital police when she was wandering naked in the hospital. She became agitated and spat and bit security guards and hospital staff. A police officer swung at her and kept swinging at her and hitting her even after she turned her back to him.^38^
2019	Doctors Hospital	Augusta, Georgia	NA	Female	NA	NA	Patient in ED became agitated after waiting for a long time, raising her voice and cursing to get nurses’ attention. A nurse and security officer responded and moved her farther away from the door where she was stationed in a wheelchair. When she attempted to stand up, security placed her in a chokehold and dragged her over the back of the chair. Patient sustained bruises and scrapes on her back.^39^
2019	St Joseph’s Hospital	Elmira, New York	49	Male	White	Y	Patient in mental health crisis was noted to have attacked an officer (although this was disputed by the family, who report he was simply wandering in the hallway). The officer subsequently struck the patient’s face against the hospital floor, pressed his foot onto patient’s neck or head area, grabbed him by his hair, and slammed his head and face into the floor additional times. The patient died.^40^
2019	Cherokee Medical Center	Gaffney, South Carolina	42	Male	White	NA	Patient who was under arrest and admitted for shortness of breath because combative while in the hospital struck the officer at his bedside. Hospital security deployed a stun gun, and then the man became unresponsive and died.^41^
2021	Charlotte-Mecklenburg Hospital	Charlotte, North Carolina	16	Male	White	Y	Mother of patient in mental health crisis asked for security officers’ help to get him inside the hospital, which led to escalation in force. Officers grabbed him by the throat, threw him to the ground, repeatedly used a stun gun, hit him in the chest, and slammed his face to the ground.^42^
**Patient arrests**							
2015	Hospital	Blountstown, Florida	57	Female	Black	NA	Patient refused to leave after being discharged, reporting continued symptoms and difficulty breathing. Staff at hospital called police who came to arrest her and escort her outside. She collapsed in the parking lot and was later noted to have a blood clot in her lungs on autopsy.^43^
2015	VA Hospital	Phoenix, Arizona	34	Male	White	NA	Patient was at the hospital and was deemed a trespasser. When police came to arrest him, he allegedly kicked officers and bit one’s thumb. He was arrested and charged.^44^
2016	Meadowland Hospital Medical Center,	Secaucus, New Jersey	29	Male	NA	NA	Patient became combative with hospital staff, prompting staff to call the police. When police arrived, the situation escalated and patient became more combative, kicking a nurse, spitting at a security guard, and throwing a headbutt at the police officer. He was arrested.^45^
2016	Baptist Health Medical Center	Little Rock, Arkansas	20	Male	Black	NA	Patient receiving treatment at a hospital shoved a hospital security guard in an altercation and was arrested.^46^
2019	Freeport Health Network Memorial Hospital	Freeport, Illinois	NA	Male	Black	NA	Patient being treated for pneumonia and asthma at the hospital was accused of stealing medical equipment when a hospital guard found him on a walk with his intravenous pole. Hospital guard called police officers to arrest the man.^47^
2019	Our Lady of the Lake Regional Medical Center	Baton Rouge, Louisiana	33	Male	NA	Y	Patient with mental illness was arrested after he slapped an emergency department employee.^48^
2021	Ben Taub Hospital	Houston, Texas	35	Male	Black	NA	Patient was arrested after he grabbed at the gun of a police officer who was escorting the patient to the bathroom, and the gun went off.^49^
**Sexual assault**							
2015	Sequoia Hospital	Redwood City, California	23	Female	NA	NA	Hospital security supervisor, who was also a police officer, lured at least 2 women into the hospital where he posed as a physician and conducted inappropriate medical examination in a utility closet. He had posted an advertisement for a modeling job and told them they needed the examination as a condition of employment.^50^
2018	St Bernard Hospital	Chicago, Illinois	NA	Male	NA	NA	Patient arrested for a misdemeanor and in the hospital undergoing a psychiatric evaluation was sexually assaulted by a police officer while cuffed to his bed. The officer grabbed the man’s penis and performed oral sex on the patient.^51^
**Hospital personnel being shot, injured or arrested**							
2017	The University Hospital	Salt Lake City, Utah	NA	Female	White	NA	Police handcuffed and arrested nurse for refusing a police request to draw blood from a patient according to hospital policy.^52^
2017	VA Hospital	Fayetteville, Arkansas	NA	Female	NA	NA	Police arrested and charged nurse with disorderly conduct and resisting arrest because she wanted to report misconduct from a VA police officer.^44^
2018	Cleveland Clinic	Cleveland, Ohio	NA	NA	Black	NA	Two employees for a vendor that supplies food to patients at the clinic were stopped and confronted, and 1 was pinned against the vehicle by hospital police.^34^
2020	Brigham and Women’s Hospital	Boston, Massachusetts	NA	NA	NA	NA	Municipal police shot a hospital valet when responding to a mentally ill man brandishing a fake gun.^53^
2020	Indiana University Health Hospital	Indianapolis, Indiana	NA	NA	Black	NA	Two janitors were accused by hospital police of making a drug deal on the job and lost their janitorial jobs. It was later determined that they were exchanging the key to a building that needed to be cleaned according to the lawsuit.^54^

Abbreviations: ED, emergency department; NA, not applicable; VA, Veterans Affairs; Y, yes.

These incidents occurred across 25 US states. Many news articles did not specify survivors’ or victims’ race and/or ethnicity (18 incidents). Among those that did, most survivors and victims were Black (19 individuals), followed by White (12 individuals). Mental illness was the most documented medical condition among patients injured or killed by health care–affiliated police and security personnel (22 patients).

### Patients Shot by Police or Security Personnel

We found that in several incidents of police or security personnel shooting patients (7 incidents), the altercations escalated because patients gained access to an officer’s gun or other weapon. Moreover, we found that the distinction between local police, private police, and hospital security personnel was often blurred. These roles regularly interfaced, cooperated, or even overlapped, with the same person adopting a different role depending on context. For example, in the incident involving a patient with bipolar who was shot in the chest during a manic state,^5^ the responsible security guards were in fact off-duty Houston police officers.

### Patients Subject to Excessive Use of Force

Even when gun shots were not involved, we found that inappropriate escalation and unnecessary use of force by hospital police or security personnel increased harm to patients. For example, officers placed a woman who was raising her voice and cursing to get the attention of nurses in a chokehold,^56^ punched a man already in restraints and handcuffs in the face,^57^ used a stun gun against a woman bound with restraints in a hospital bed,^5,58^ and tackled a man causing traumatic brain injury and eventual death over driving the wrong way in a hospital parking lot.^35^ We found an incident involving a minor, a 16-year-old in mental health crisis, who was beaten by security guards, including being grabbed by the throat, slammed onto the ground, and shot with a stun gun.^42^

### Patients Arrested Rather Than Adequately Treated

In other incidents, inappropriate escalation involved arrest rather than treatment for a patient’s medical condition. For example, 1 man with paranoid schizophrenia and bipolar disorder came to the emergency department seeking medical care,^48^ but he was instead arrested after slapping an emergency department employee. In another incident, a woman was arrested for refusing to leave her room after discharge as she continued to report symptoms of shortness of breath. She was arrested and escorted outside, where she collapsed, died, and was later found on autopsy to have had a blood clot in her lungs.^43^

### Patients Subject to Sexual Assault

We found 2 incidents involving security personnel sexually assaulting, which involved using their hospital privileges to gain access to the patient and/or a private room to perpetrate their assault. In 1 incident, at least 2 survivors were identified.^50^ In the other, a police officer assaulted a patient, including performing oral sex, while the patient was handcuffed to his bed.^51^

### Hospital Personnel or Others Shot, Injured, or Arrested

We found that health care personnel were also involved with incidents with law enforcement in health care facilities. In the majority of instances (10 reports), health care personnel facilitated harm inflicted by law enforcement by calling upon them to respond to patients perceived as combative. However, they were also collateral damage to security personnel actions. In 2 incidents, nurses were arrested for not following police officer orders. Those in lower-level roles were harmed more directly. In 1 incident, a valet outside of a Boston hospital was shot and killed as collateral damage.^53^ In another, 2 Black janitors in an Indiana hospital were subjected to racial stereotypes and lost their jobs after being falsely accused of making a drug deal when they had simply been exchanging a key to a building requiring cleaning.^54^

In the previously mentioned incident involving the man arrested while visiting his newborn in the neonatal unit, the patient harmed by the physical violence was the newborn who sustained a skull fracture amid the altercation with the police.^55^ Another incident involved officers restraining a daughter who was attempting to advocate for her father who had been struck by police when he attempted to leave the hospital.^28^ In this way, individuals harmed include collateral damage or individuals, whether other hospital personnel or family members, not directly party to the interaction with the security personnel.

## Discussion

Using a media content and thematic analysis, we characterized the harms reported to have been perpetuated by health care organization reliance on police and security personnel. These harms included potential death, injury, unnecessary violence, sexual assault, and arrest of patients, families, and health care staff. Our findings suggest that Black patients and patients with mental illness may be particularly vulnerable in encounters with law enforcement in health care settings, which has been well documented in the community more broadly.^59^ By linking themselves to the carceral system that has been shown to produce and sustain racial inequality, health care organizations have potential to become yet another sector or system in society that upholds inequalities along racial lines. Here, we understand carceral system as referring broadly to institutions, practices, and punitive orientations that subject people to surveillance and threat of punitive policies, thereby encompassing actions of police and security broadly and not limited to prisons and jails. That policing practices reified in health care organizations may yield the same results as they do in the community is not surprising, and in fact is emblematic of how structural racism functions to produce inequities across sectors. At the same time, racist elements to overdiagnosis of mental illnesses such as schizophrenia have been documented,^60^ suggesting that policing in hospitals that involve those with mental illness may also result in racially disproportionate harms.

The omission of data on policing in hospitals, necessitating the use of media content for this study, is itself a striking example of how structural racism is manifested in hospitals because collecting such data has not been a priority for health care organizations. Researchers, therefore, are unable to systematically track hospital organizations’ financial investments in carceral systems or potential racial disparities in the treatment of patients by hospital police or security.

We also found that harms came about due to the presence of weapons on security personnel rather than a patient being armed. In a 2012 study reviewing hospital-based shootings from 2000 through 2011, about 23% of emergency department shootings involved someone grabbing a gun from security personnel.^61^ Although it is well accepted that the availability of weapons increases the risk of homicide and suicide in patients’ homes,^62,63^ health care systems have not applied the same knowledge to decrease the risk of violence in hospitals by disarming security personnel. Notably, the use of tasers in hospitals, often touted as a safer weapon alternative, also led to deaths and other physical harms. There is no evidence in the broader literature that they mitigate violence-related injury among staff.^64^

Because structural racism shapes social relations beyond any one sector such as health care, it is important to recognize that increased interactions with private security occurs across sectors. Thus, health care organizations may play a role in a cumulative experience of harm for structurally vulnerable individuals, especially as interactions with law enforcement in daily life are associated with psychological harms.^65^ In 1 study of patients with drug use, for example, participants reported experiencing surveillance across multiple social spaces in their daily lives, including but not limited to malls, stores, pharmacies, government offices, public parks, sidewalks, and public transport systems.^66^ Structurally vulnerable individuals, disproportionately from communities of color, are more likely to be subject to these forms of sociospatial control because they lack access to private spaces and are therefore more likely to rely on public or private organization property for everyday activities.

Potential policy solutions include eliminating armed security personnel, eliminating the use of police officers as security personnel, and avoiding arrests in health care settings. We recognize that violence against health care staff presents a major challenge to safety—1 systematic review found that 62% of health care workers reported exposure to any form of workplace violence, with 24% reporting experiencing physical violence.^67^ Some health care workers may assume that police or security personnel confer safety and can address this challenge. But this assumption is not backed by evidence. Moreover, health care workers may have differing perceptions of safety offered to them by police, who have traditionally not offered protection equally across racial lines. In other words, white health care workers who may be beneficiaries of positive interactions with, and protection by, the police may be more supportive of their presence in health care settings than those from marginalized racial groups. Further conversations are also needed about what degree of force, if any, may be justifiable in health care settings. There may be different ethical, moral, medical, and legal stances to reconcile within the profession and society at large.

At the same time, there is growing evidence that racial biases are manifested when clinical staff justify patient monitoring or intervention through physical restraints or psychopharmacology; that is, we see differential application of physical restraints among Black patients relative to others.^68^ In fact, study after study has revealed clinicians equally embracing societal biases, including regarding race,^69^ mental illness,^70,71^ and disability.^72^ Organizational approaches to addressing violence against health care workers should therefore remain cognizant and vigilant of these biases to mitigate them. One approach can include using behavioral response teams to respond to agitated patients or those in mental health crises instead of security personnel.

To better understand the scope of the harms highlighted in our study, implementing clear transparency and accountability standards for security personnel inclusive of data collection and release of policing and security practices in hospitals must be a priority. Just as health care organizations release health equity dashboards, information about policing and security practices could be integrated into them and include information about when security personnel are called, by whom, for whom, and potentially even linked to patient electronic health records to better understand differences across demographic and clinical characteristics.^73^ Key elements of successful dashboards include actively reaching and engaging stakeholders,^73^ so we encourage involving health care workers, community-based organizations, and patient advocates in such an effort.

Health care organizations should also consider instituting policies and training for health care staff on interactions with law enforcement, especially as clinicians and police have conflicting responsibilities. Clinicians are dedicated to promoting individual well-being, whereas policing has often been reported to perpetuate the harms hospitals are designed to heal. This conflict has been recognized by patients who report distrust of clinicians and health care organizations due to real or perceived involvement with the carceral system,^74,75,76^ making health care organizations’ explicit guidance on interactions with law enforcement paramount. This should include clearly distinguishing roles and expectations of municipal police vs hospital police or security personnel, who we found often overlapped and worked in concert despite potential key professional duties.

Notably, our focus on health care organizations and institutional policies does not absolve individual health care professionals of harm, just as focusing on structural racism and related policies such as Jim Crow or redlining does not absolve an individual engaging in acts of racism such as cross-burning. Individuals perpetuating racial inequality are often empowered by membership and organizational roles.^77^ After all, these roles provide legitimating access to rules and resources that, when applied, can increase (or decrease) racial inequality. In the context of hospital police or security personnel, for example, we found that security teams often appeared in response to clinicians’ expression of security need. Prior studies have demonstrated that health care personnel’s perceptions of risk of potential harm from a patient likely vary with the patient’s race.^78,79^ For example, the 26-year-old student with bipolar disorder who was shot in the chest by off-duty police officers was summoned by a nurse after the patient refused to fasten his patient gown; it is conceivable that the nurse may not have contacted the police had the patient not been a young Black male.

Health care staff can also serve as agents of law enforcement, conducting diagnostic tests solely for law enforcement purposes (eg, mandatory blood testing or scans),^80,81,82^ differentially referring Black vs White women who use drugs during pregnancy to law enforcement^83,84^ and differentially referring Black vs White families to child protective services.^84^ This represents another manifestation of a racialized carceral logic that attributes criminality and blame to people of color, exacerbating the unequal outcomes of policing in health care settings. Indeed, incorporating carceral practices into health care systems—be they arrest, criminal charges and investigation, or ultimately, incarceration—all negatively affect an individual’s life trajectories,^85,86,87^ reducing their agency and perpetuating health inequities.

### Limitations

This study has some limitations. First, since there is no systematic collection of policing practices in health care settings, the study was limited to searchable online news media and subject to publication bias and differences in journalistic content across articles (ie, whether race and ethnicity of individuals involved or details about their medical or mental health history were included). We cannot make claims about how representative these policing practices are as there are no data to understand the denominator, or the total encounters between law enforcement in health care settings and patients or staff, and whether they result in harm. Collecting systematic data on policing in hospitals is hence one of many policies we recommend to further understand and address the harm caused by the conflation between the health care and carceral systems. Second, our study design does not capture health harms of security personnel that do not result from violence. For example, security guards have been found to limit people’s access or entry into health care spaces (eg, a patient with slurred speech who had a stroke but was perceived to be drunk and turned away).^66^ In this way, health care–affiliated security personnel can be associated with health at various points in someone’s life, from their daily interactions within and outside health care organizations, which future studies should further elucidate.

## Conclusions

Health care organizations are supposed to be places of healing. Allowing harrassment or physical harm of patients and staff undermines this mission. Because carceral systems disproportionately target racially marginalized groups, they also play a role in racialized health inequalities. By bringing attention to the practice of police and security personnel in health care settings, we seek to facilitate a pathway for health care organizations to live up to their stated missions as healing organizations. The changes we suggest are needed not only to limit abuses but also to improve public health.

## Notes

Supplement.
Data Sharing Statement
