# Supplementary material for: Police Violence in Health Care Settings in US Media Coverage
Source: JAMA Netw Open. 2023 Nov 13;6(11):e2342998. doi: 10.1001/jamanetworkopen.2023.42998 (PMC10644214; doi:10.1001/jamanetworkopen.2023.42998)
Supplement: Supplement. — Data Sharing Statement [file jamanetwopen-e2342998-s001.pdf]

Data Sharing Statement

Saadi. Police Violence in Healthcare Settings in US Media Coverage. *JAMA Netw Open*. Published November 13, 2023. doi:10.1001/jamanetworkopen.2023.42998

Data
Data available: No

Additional Information
Explanation for why data not available: All data is already made available in the manuscript.
